# Supplementary material for: In Vitro and In Vivo Activity of 14-O-[(4,6-Diamino-pyrimidine-2-yl) thioacetyl] Mutilin against Methicillin-Resistant Staphylococcus aureus
Source: Molecules. 2021 May 28;26(11):3277. doi: 10.3390/molecules26113277 (PMC8199141; doi:10.3390/molecules26113277)
Supplement: Supplementary file 1 [file molecules-26-03277-s001.zip › molecules-1192818-supplementary.pdf]

Supplementary data

In vitro and In vivo Activity of 14-O-[(4,6-Diamino-pyrimidine-2-yl) thioacetyl] Mutilin Against Methicillin-Resistant *Staphylococcus aureus*

Yunxing Fu [1,2], Chunqing Leng [3], Yuan Fan [4], Xia Ma [1], Xianghui Li [1], Xuefei Wang [1], Zhenghuan Guo [1] and Xiujun Wang [1,*], Ruofeng Shang [4,*]

[1] Zhengzhou Key Laboratory of Immunopharmacology of effective components of Chinese Veterinary Medicine, College of Veterinary Medicine, Henan University of Animal Husbandry and Economy, 450046 Zhengzhou, PR China; fyx1261648623@163.com (Y.X.); maxia801010@126.com (X.M.); huiskys@163.com (X.L.); hnmywxf001@126.com (X.W.); 80791@hnuahe.edu.cn (X.Wa)

[2] Henan Provincial Research Center for the inheritance and innovation of Chinese veterinary medicine classic prescriptions, College of Veterinary Medicine, Henan University of Animal Husbandry and Economy, 450046 Zhengzhou, PR China

[3] Department of Animal Production, Jinhua Polytechnic, 321000, Jinhua, PR China; 20191009@jhc.edu.cn

[4] Key Laboratory of Veterinary Pharmaceutical Development, Ministry of Agriculture and Rural Affairs, Lanzhou Institute of Husbandry and Pharmaceutical Sciences of CAAS, 730050 Lanzhou, PR China

* Correspondence: wxjfly@126.com (X.W.); shangrf1974@163.com (R.S.)

Table of Contents

Figure S1. IR, ^{1}H-NMR and ^{13}C-NMR Spectra of DPTM……………………………………………………1-3

Table S1. MIC values of DPTM and Tiamulin fumarate against clinical isolates of MRSA……………………...4

Figure S1. IR, ^{1}H-NMR and ^{13}C-NMR Spectra of DPTM

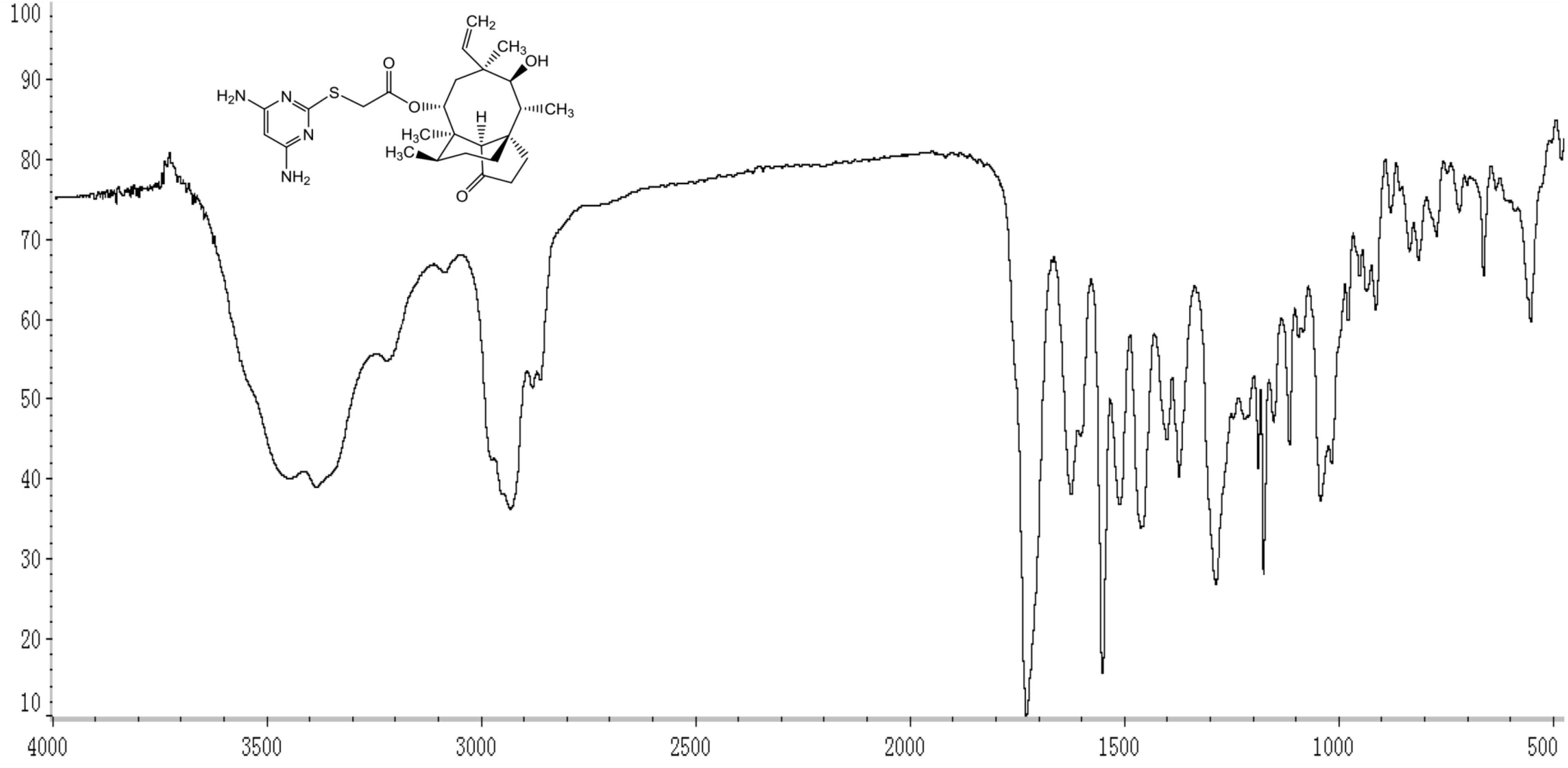

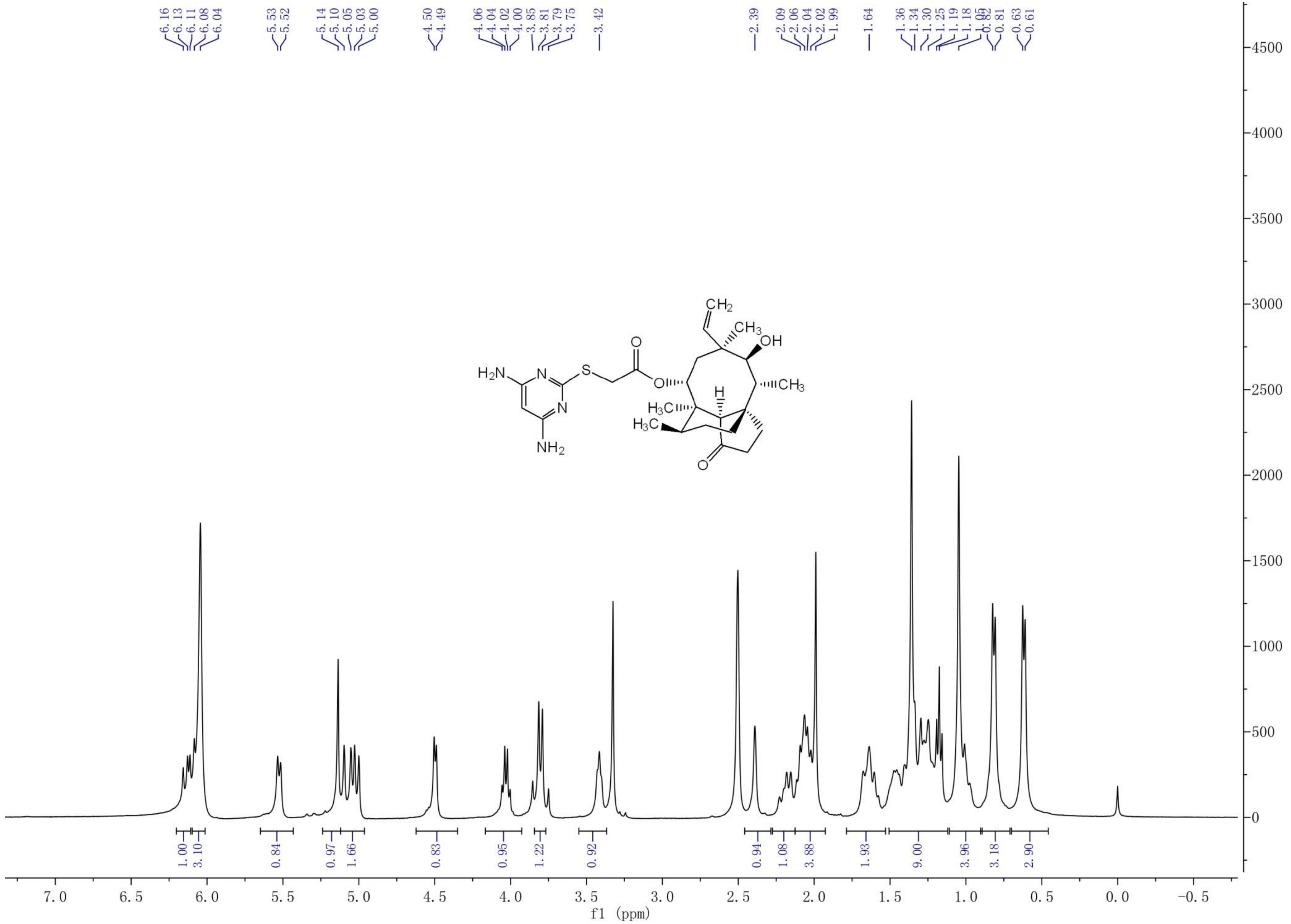
6.16
6.13
6.11
6.08
6.04
5.53
5.52
5.14
5.10
5.05
5.03
5.00
4.50
4.49
4.06
4.04
4.02
4.00
3.85
3.81
3.79
3.75
3.42
2.39
2.09
2.06
2.04
2.02
1.99
1.64
1.36
1.34
1.30
1.25
1.19
1.18
1.05
0.82
0.81
0.63
0.61
CH2
CH3
OH
O
H2N
N
S
H
H3C
NH2
1.00
3.10
0.84
0.97
1.66
0.83
0.95
1.22
0.92
0.94
1.08
3.88
1.93
9.00
3.96
3.18
2.90
7.0
6.5
6.0
5.5
5.0
4.5
4.0
3.5
3.0
2.5
2.0
1.5
1.0
0.5
0.0
-0.5
f1 (ppm)
4500
4000
3500
3000
2500
2000
1500
1000
500
0

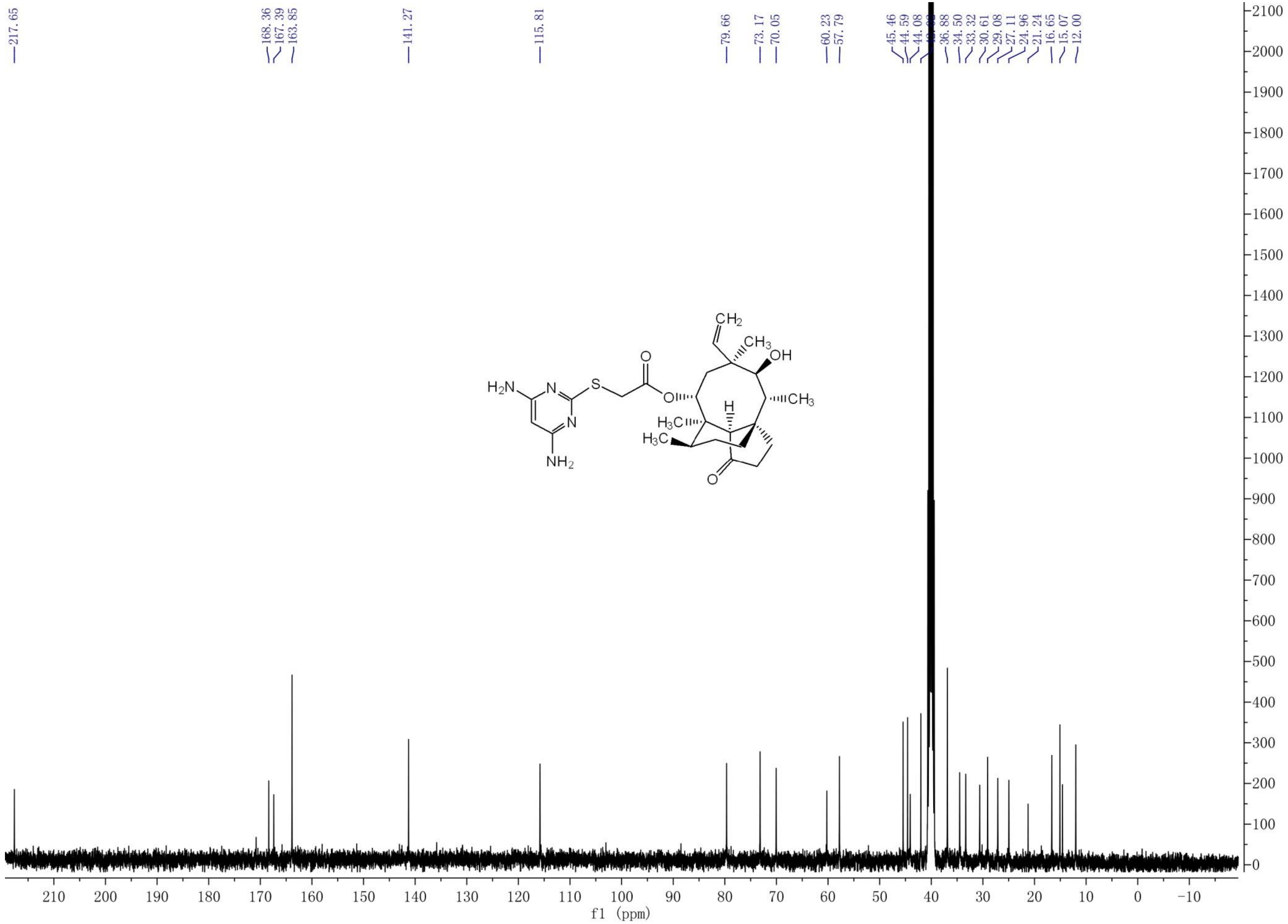
217.65
168.36
167.39
163.85
141.27
115.81
79.66
73.17
70.05
60.23
57.79
45.46
44.59
44.08
36.88
34.50
33.32
30.61
29.08
27.11
24.96
21.24
16.65
15.07
12.00
f1 (ppm)

Table S1. MIC values of DPTM and Tiamulin fumarate against MRSA strains.

Stain[a]	SCCmec[b]	MIC (μg/mL)		Stain[a]	SCCmec[b]	MIC (μg/mL)	
		DPTM	Tiamulin			DPTM	Tiamulin
MRSA-21	III	0.0625	0.25	MRSA-75	II	0.25	0.5
MRSA-23	III	0.0625	0.125	MRSA-79	II	0.0625	0.125
MRSA-24	IV	0.125	0. 5	MRSA-85	II	0.0625	0.25
MRSA-32	IV	0.125	0.25	MRSA-90	IV	0.125	0.25
MRSA-33	IV	0.0625	0.125	MRSA-94	III	0.0313	0.125
MRSA-38	I	0.0625	0.125	MRSA-99	III	0.0625	0.125
MRSA-39	II	0.25	1	MRSA-108	II	0.0625	0.25
MRSA-40	I	0.0625	0.125	MRSA-122	I	0.0625	0.25
MRSA-44	I	0.0625	0.25	MRSA-128	IV	0.0313	0.125
MRSA-45	II	0.313	0.125	MRSA-133	IV	0.125	0.25
MRSA-47	III	0.125	0.5	MRSA-135	III	0.25	0.5
MRSA-48	II	0.125	0.25	MRSA-141	III	0.125	0.25
MRSA-51	II	0.25	1	MRSA-147	II	0.0625	0.125
MRSA-53	II	0.0625	0.25	MRSA-149	III	0.125	0.5
MRSA-54	IV	0.0625	0.5	MRSA-153	III	0.0625	0.5
MRSA-55	IV	0.125	0.25	MRSA-155	III	0.0625	0.25
MRSA-57	IV	0.0625	0.125	MRSA-157	IV	0.0625	0.25
MRSA-59	III	0.0625	0.25	MRSA-159	II	0.0625	0.5
MRSA-60	III	0.0313	0.125	MRSA-160	IV	0.0625	0.5
MRSA-61	I	0.0625	0.5	MRSA-166	IV	0.0625	0.25
MRSA-64	IV	0.0625	0.125	MRSA-173	II	0.125	0.25
MRSA-67	III	0.0625	0.25	MRSA-177	III	0.125	0.5
MRSA-68	III	0.125	0.25	MRSA-180	III	0.25	1
MRSA-70	IV	0.25	0.5	MRSA-185	II	0.0625	0.125
MRSA-71	III	0.0625	0.125	MRSA-188	IV	0.125	0.125
MRSA-72	III	0.0625	0.25	MRSA-194	IV	0.0625	0.125
MRSA-73	II	0.125	0.125	MRSA-197	III	0.125	0.5

[a]Strains were collected from dairy farms located in Gansu province, China.

[b]Staphylococcal cassette chromosome mec types.
